# The mixed layer modified radionuclide atmospheric diffusion based on Gaussian model

**DOI:** 10.3389/fpubh.2022.1097643

**Published:** 2023-01-04

**Authors:** Ting Li, Xiaolei Zheng, Shengpeng Yu, Jin Wang, Jie Cheng, Jie Liu

**Affiliations:** ^1^Hefei Institutes of Physical Science, Chinese Academy of Sciences, Hefei, Anhui, China; ^2^University of Science and Technology of China, Hefei, Anhui, China; ^3^International Academy of Neutron Science, Qingdao, Shandong, China; ^4^China Three Gorges University, Yichang, Hubei, China

**Keywords:** radionuclide, atmospheric diffusion, inversion temperature, mixed layer, Gaussian diffusion model

## Abstract

**Background:**

Atmospheric diffusion is often accompanied by complex meteorological conditions of inversion temperature.

**Methods:**

In response to the emergency needs for rapid consequence assessment of nuclear accidents under these complex meteorological conditions, a Gaussian diffusion-based model of radionuclide is developed with mixed layer modification. The inhibition effect of the inversion temperature on the diffusion of radionuclides is modified in the vertical direction. The intensity of the radionuclide source is modified by the decay constant.

**Results:**

The results indicate that the enhancement effect of the mixed layer on the concentration of radionuclides is reflected. The shorter the half-life of the radionuclide, the greater the effect of reducing the diffusion concentration. The Kincaid dataset validation in the Model Validation Kit (MVK) shows that, compared to the non-modified model, predictions of the modified model have an enhancement effect beyond 5 km, modulating the prediction values to be closer to the observation values.

**Conclusions:**

This development is consistent with the modification effects of the mixed layer. The statistical indicators show that the criteria of the modified model meet the criteria of the recommended model.

## 1. Introduction

There are a large number of nuclear facilities, such as nuclear power plants and nuclear fuel recycling plants, around us. Accidents, such as fire or explosion, may lead to leakage and diffusion of radionuclides from these nuclear power plants and nuclear fuel recycling plants (1, 2). As regards the radioactive waste landfill in Westlake, Missouri, federal scientists have predicted that the spread of radioactive material caused by an underground fire near the waste repository could kill residents of the state (3). Also, terrorist attacks using dirty bombs may cause radioactive contamination at the risk of public health (4, 5). The International Commission on Radiological Protection (ICRP) research shows that threats to the public are most likely to come from terrorist attacks using radioactive material, such as the dirty bomb explosion (6). Professor Graham Allison of the Harvard Kennedy School predicted that the probability of a nuclear terrorist attack would increase significantly in the 10 years after 2015 (7).

The Gaussian model was more widely adopted in the simulation of atmospheric diffusion in terms of rapid consequence assessment of nuclear accidents (8). To get a direct understanding of the affected areas of events consequent to nuclear and radiological terrorism events in city areas, Luo Lijuan developed a new software system based on the Gaussian diffusion model to predict the spread and deposition of radioactive pollutants (9). To carry out the preliminary analysis of a gas leakage accident simulation of China Lead-based Research Reactor (CLEAR-I), a Gaussian plume model with Pasquill-Gifford dispersion parameters was used for analyzing atmospheric transport and dispersion of radioactive material (10). Hyo-Joon Jeong conducted a study on the dispersal of radionuclides of terrorist attacks using radioactive material in public areas and used a Gaussian model to simulate the diffusion of radionuclides into the atmosphere (11). To rapidly assess the effect of radioactive materials on public health, Bo Cao developed the Radionuclide Atmosphere Dispersion Codes (RADC) with the FORTRAN language based on the Gaussian diffusion model (12, 13). Visscher applied Gaussian models to industrial terrains and preliminarily corrected the distribution effect of wind blowing from these industrial terrains on diffusion (14).

However, these Gaussian models of radioactive atmospheric diffusion were relatively simple in considering the atmospheric boundary layer. The inhibition effect of the mixed layer formed by the phenomenon of inversion temperature on the diffusion of radionuclides was not considered. The atmospheric stability was determined to be stable, neutral, or unstable, and the diffusion coefficient was obtained according to the empirical formula. Therefore, under the complex meteorological conditions of inversion temperature, the Gaussian atmospheric diffusion model was insufficient to simulate accurately the atmospheric diffusion of radionuclides. In this study, a novel method for carrying out fast atmospheric diffusion of radionuclides based on the Gaussian diffusion model was developed using the height modification of the mixed layer.

## 2. Materials and methods

### 2.1. Conventional Gaussian diffusion model

The Gaussian model is derived from the turbulent diffusion equation, in which the diffusion coefficient K is given as a constant. The spatial radionuclide concentration formula for the elevated source emission is given as follows, where the first term in the curly bracket represents the contribution of the mirror source and the second term in the curly bracket represents the contribution of the elevated source (15).


(1) $$\begin{array}{l} C\left(x,y,z,h\right)=\frac{\dot{Q}}{2\pi u\sigma_{y}\sigma_{z}}\exp\left(-\frac{y^{2}}{{{2\sigma }_{y}}^{2}}\right)\cdot \{\exp\left[\frac{-{\left(z-h\right)}^{2}}{{{2\sigma }_{z}}^{2}}\right]\\ \;+\exp\left[\frac{-{\left(z+h\right)}^{2}}{{{2\sigma }_{z}}^{2}}\right]\} \end{array}$$

where *C*(*x, y, z*) indicates the average concentration of air pollutants at the point (*x, y, z*) in Bequerel per cubic meter (Bq/m^3^). $\overset{\cdot }{Q}$ is the source intensity, indicating the release rate of radionuclide activity, in Bequerel per second (Bq/s).

σ_*y*_, σ_*z*_ are the functions of distance in the downwind direction x(m), representing the standard deviation of the normal distribution of plume concentrations in the crosswind direction (y) and vertical direction (z), respectively, in m. The diffusion coefficients in the y and z directions are given as functions of the stability classes of the atmosphere according to Pasquill-Gifford (16).


(2) $$\sigma_{y}=ax^{b+clnx}$$

(3) $$\sigma_{z}=dx^{e+flnx}$$

where *a, b, c* of Equation (2) and *d, e, f* of Equation (3) are determined by the stability class of the atmosphere.

### 2.2. Radionuclide diffusion modified model

The atmospheric diffusion process of radionuclides is affected jointly by the wind field, the underlying surface, and the interaction between them. These three factors determine the radionuclide transportation, diffusion, and their dilution into the atmosphere (17, 18).

Atmospheric diffusion is often accompanied by complex meteorological conditions of inversion temperature. The air in the inversion temperature layer is heavy on the top and light on the bottom in the vertical direction. The atmospheric layer in which the temperature increases with height in the vertical direction is called the mixed layer, which inhibits the diffusion of atmospheric pollutants in the upward direction (19, 20). The atmospheric diffusion of radionuclides under complex meteorology in this area is different from those of other areas. During the atmospheric diffusion of short-lived radionuclides, they decay and transform into other radionuclides over time, reducing the total amount of radionuclides in the atmosphere.

**Figure 1:**
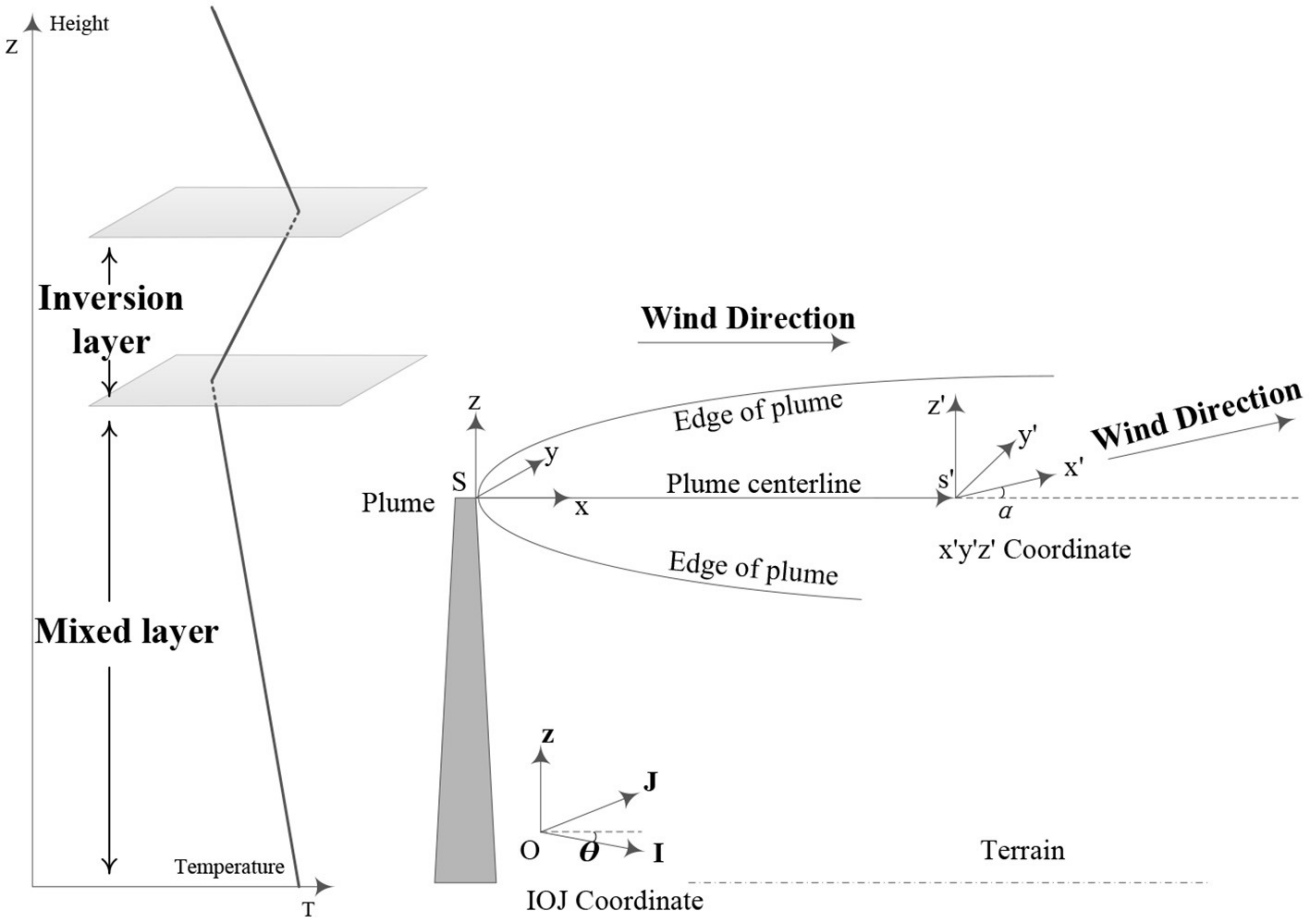
Schematic diagram showing the mixed layer modified atmospheric diffusion model of radionuclide under complex meteorological conditions.

As a result, taking into account the mixed layer and radionuclide decay, a Gaussian radionuclide atmospheric modified diffusion model based on the mixed layer was established to assess the radionuclide diffusion consequence quickly. The radionuclide diffusion model under complex meteorological conditions is established, as shown in Figure 1.

#### 2.2.1. Mixed layer modification

**Figure 2:**
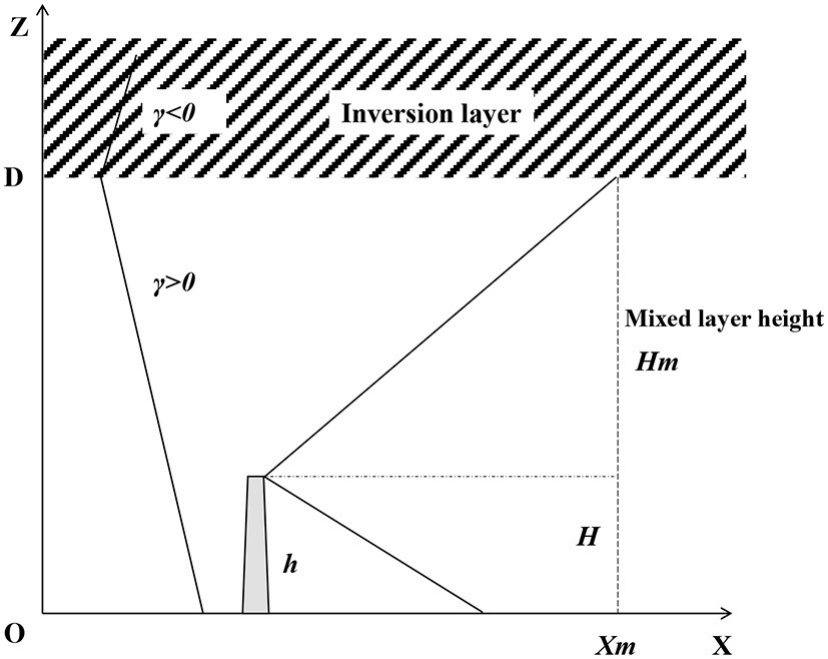
Schematic diagram showing the mixed layer modified atmospheric diffusion.

A schematic diagram showing the mixed layer modified atmospheric diffusion model of radionuclides under complex meteorological conditions is shown in Figure 2. The lower air constitutes the mixed layer, and the upper air constitutes the inversion layer. γ is the height of the mixed layer. γ is the vertical decrement rate of air temperature, expressed as γ = –dT/dZ. γ < 0 means the air temperature increases with the increase in height, which corresponds to the appearance of inversion temperature.

The diffusion of radionuclides into the mixed layer is limited to the space between the ground and the inversion layer. The radionuclide diffusion concentration shows the combined result of the chimney source and the mirror source from the ground and the top surface of the mixed layer.

The vertical diffusion coefficient is given as ${\sigma_{z}}^{m},$ where the upper bound of the radionuclide diffusion plume meets the bottom bound of the inversion temperature layer. At this point, *C*(*x*_*m*_, 0, *H*_*m*_, *h*) is close to 0. According to the atmospheric stability and diffusion coefficient, the downwind distance *x*_*m*_ corresponding to ${\sigma_{z}}^{m}$ is calculated by Equations (1)–(3).

The downwind distance *x* is divided into three parts.

1. When the downwind distance *x* ≤ *x*_*m*_, the diffusion of radionuclides is not affected by the inversion temperature layer, and the concentration of radionuclides *C* is still calculated according to Equation (1).
2. When the downwind distance *x* ≥ 2*x*_*m*_, the diffusion of radionuclides is reflected multiple times by the upper boundary of the mixed layer and the ground, so the radionuclide concentration distribution in the vertical direction is close to uniform.
(4) $$C\left(x,y,z\right)=\frac{\dot{Q}}{\sqrt{2\pi }uH_{m}\sigma_{y}}\exp\left(-\frac{y^{2}}{{{2\sigma }_{y}}^{2}}\right),$$
  where *C*(*x, y, z*) indicates the average concentration of radionuclides at the point (*x, y, z*), given in *Bq*/*m*^3^. $\overset{\cdot }{Q}$ is the source intensity, indicating the release rate of radionuclides, given in *Bq*/*s*. *H*_*m*_ represents the standard deviation of the plume concentration normal distribution in the crosswind direction (y), given in m. σ_*y*_ indicates the average wind speed, given in *m*/*s*. *H*_*m*_ indicates the height of the mixed layer, given in m.
3. When the downwind distance *x* is between *x*_*m*_ and 2*x*_*m*_, the radionuclide concentration *C* is obtained by the logarithmic interpolation of *C* at the two points *x*_*m*_ and 2*x*_*m*_.

#### 2.2.2. Radioactive decay modification

As regards the decay of radionuclides, it was observed that they get transformed into other radionuclides over their diffusion into the atmosphere.

The reduction of radionuclides in the diffusion process conforms to the exponential decay law, so the source intensity $\overset{\cdot }{Q}$ can be modified by the decay constant.


(5) $$\overset{\cdot }{Q}(x)=\overset{\cdot }{Q}\exp\;(-\frac{\lambda_{r}x}{u})\;,$$

where λ_*r*_ represents the radionuclide decay constant, given in /*s*. *u* indicates the wind speed, given in meters per second (m/s).

### 2.3. Model Validation Kit

The National Environmental Research Institute (NERI) of Denmark has developed a model validation toolkit MVK for quantitative evaluation by mathematical statistics, including Kincaid experimental datasets of atmospheric diffusion.

The Kincaid dataset was named after the tracer experiment of the Kincaid Power Plant. The chimney height is 180 meters. The detectors are arranged in the interval of 0.5–50 km, which is 0.5, 1, 2, 3, 5, 7, 10, 15, 20, 30, 40, and 50 km, respectively, with a total of 12 parts. Meteorological data, such as the mixed layer, wind speed, and wind direction, can be found on the web page of the initiative on Harmonization within Atmospheric Dispersion Modeling for Regulatory Purposes (21). The observed mixing heights were determined manually by the interpretation of radiosonde data. MVK was used to validate the simulation results of the modified atmospheric diffusion model with 586 sets of data with quality indices of 2 and 3 in the Kincaid dataset. The simulated area was 80 × 80 km.

The MVK has on hand various statistical and analysis tools to integrate the quantitative analysis program (BOOT). The statistical indicators include the mean value (mean) and the standard deviation (Sigma). The mean deviation can measure only the difference between the average levels of the two sets of data, and the indicator alone cannot indicate the model's overall performance. BOOT also gives fraction bias, mean square error, correlation coefficient, and proportional factor 2. The expressions and ideal values of each statistical index are given in Table 1. Fractional bias (FB), non-modified model mean square error (NMSE), and Factor of 2 observations (FA2) are used to represent these indicators.

**Table 1:** Statistical indicators of the BOOT analysis program.

**Statistical indicator**	**Formula**	**Ideal value**
*FB*	$FB=\frac{\bar{C_{o}}-\bar{C_{p}}}{0.5\left(\bar{C_{o}}+\bar{C_{p}}\right)}$	0.0
*NMSE*	$NMSE=\frac{\bar{{\left(C_{o}-C_{p}\right)}^{2}}}{\bar{C_{o}}\bar{C_{p}}}$	0.0
*FA*2	$FA\alpha =\frac{N\left(y-y_{0}=\left(x-x_{0}\right)\times 2\right)}{N}$	1.0

Among them, *C*_*o*_ represents the observation value, *C*_*p*_ represents the model calculated value, σ_*o*_ is the standard deviation of the observation value, σ_*p*_ is the standard deviation of the model calculated value, and the unit of each quantity is μ*g*/*m*^3^.

### 2.4. Criteria of the recommended model

According to Chang and Hanna (22), the statistical indicators satisfy the conditions of −0.3 < FB < 0.3, NMSE < 1.5, and FA2 > 0.5, indicating that the prediction model has a relatively high reliability.

## 3. Results and discussion

First, the atmospheric diffusion-modified model of radionuclides is verified, and the effects of the mixed layer and radionuclide decay on radionuclide diffusion concentration are studied and the results are analyzed. Then, the MVK (Model Validation Kit) is introduced to validate the modified model to evaluate its prediction performance with Kincaid experimental datasets.

### 3.1. Verification of the modified model

The typical radionuclide I-131 is selected for atmospheric diffusion. The release rate of the radionuclide is 1 × 10^5^ Bq/s, the chimney height is 180 m, the average wind speed is 2.9 m/s, and the dominant wind direction is west.

#### 3.1.1. The effect of mixed layer on radionuclide concentration

**Figure 3:**
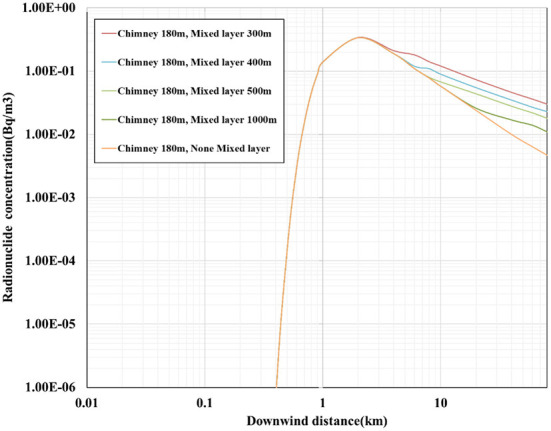
The effect curve of mixed layer on the concentration of radionuclides.

The curve of the effect of the mixed layer on the concentration of radionuclides is shown in Figure 3. The height of the mixed layer is set as 300, 400, 500, and 1,000 m, respectively, and the concentration of radionuclides is calculated in the range of 60 km.

Figure 3 shows that the curve depicting the concentration of radionuclides in the downwind direction can be divided into two parts, namely the rising part at a short distance and the descending part at a long distance. In the dividing point, the maximum concentration of radionuclides appears in the downwind direction.

When the maximum value of the concentration of radionuclides in the downwind direction becomes smaller, the maximum value reached is 3.4 × 10^−1^ Bq/m^3^, and the maximum value appears at 2 km. It shows that, when the chimney height increases, the maximum value of the concentration of radionuclides in the downwind direction decreases, and the distance from the maximum value in the downwind direction increases. When the height of the mixed layer is 300 m, the concentration of radionuclides in the downwind direction begins to increase at 4 km. When the height of the mixed layer is 400 m, the concentration of radionuclides in the downwind direction begins to increase at 5 km, and when the height of the mixed layer is 500 m, the concentration of radionuclides in the downwind direction begins to increase at 8 km. It is explained using the fact that the radioactivity contamination is exacerbated by the low height of the mixed layer due to the low vertical inversion temperature.

The upward diffusion of the radionuclide plume encounters the low boundary of the mixed layer and reflects the downwind diffusion. Compared with none mixed layer modification, the reflected plume contributes additionally to the concentration of radionuclides in the downwind direction, resulting in a larger downwind concentration.

#### 3.1.2. The effect of radionuclide decay on the concentration of radionuclides

I-131 isotopes and half-life describing the effect of radionuclide decay on the concentration of radionuclides are listed in Table 2, including I-122, I-118, I-128, I-132, and I-129 isotopes. For a comprehensive comparison, a supposed radionuclide with a very short half-life of 30.8 s is selected for comparison.

**Table 2:** I-131 isotopes and half-life on the effect of radionuclide decay.

**Radionuclide**	**Half life**	**Unit**
I-122	3.62E+00	Minute
I-118	1.37E+01	Minute
I-128	2.50E+01	Minute
I-132	2.30E+00	Hour
I-129	1.57E+07	Year

**Figure 4:**
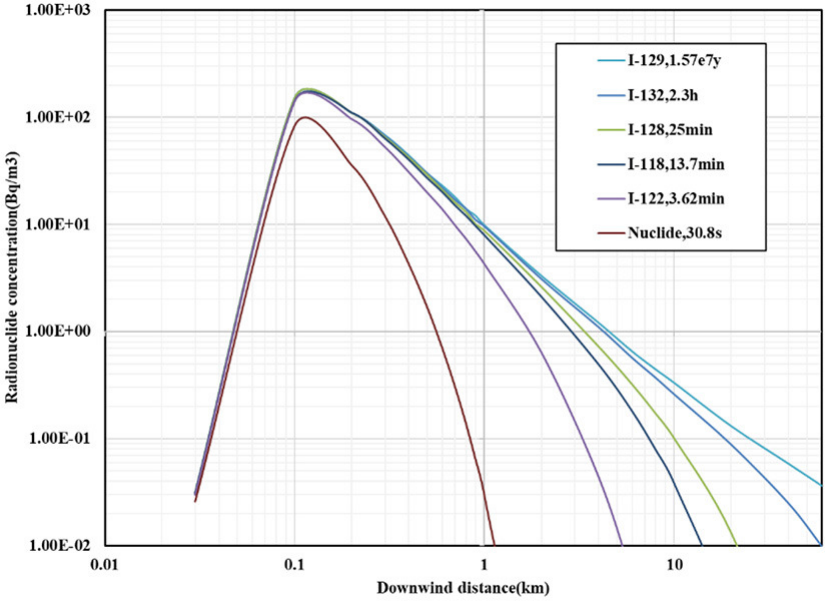
The curve of the effect of radionuclide decay on the concentration of radionuclides.

The curve of the effect of radionuclide decay on the concentration of radionuclides within 60 km is shown in Figure 4.

From the analysis of the effect of radionuclide decay on the concentration of radionuclides, it becomes known that the concentration of radionuclides in the downwind direction first increases to the maximum value and then shows a downward trend.

As regards the modified model with radionuclide decay, the downwind distance corresponding to the maximum concentration of radionuclides in the downwind direction is the same, that is, about 100 m. However, the maximum concentration of radionuclides of the modified model is 8.3 × 10^1^ Bq/m^3^, which is lower than the value of 1.5 × 10^2^ Bq/m^3^ of the non-modified model. The smaller the half-life relative to the diffusion time, the greater the effect of the decay modification on the concentration of radionuclides in the downwind direction. The radionuclide decay affects the maximum concentration of radionuclides in the downwind direction, but it does not affect the downwind distance corresponding to the maximum concentration of radionuclides.

Among the effects of the mixed layer and radionuclide decay on the concentration of radionuclides, for the chimney height of 180 m, the enhancement effect of the mixed layer on the concentration of radionuclides is reflected beyond 4 km. The decay of radionuclides has a great influence on radionuclides with a small half-life and has almost no effect on radionuclides with a longer half-life.

### 3.2. Validation of the modified model

The Model Validation Kit (MVK) has been widely used and has become the main tool for evaluating and validating atmospheric diffusion models.

#### 3.2.1. Plot analysis of simulation results

**Figure 5:**
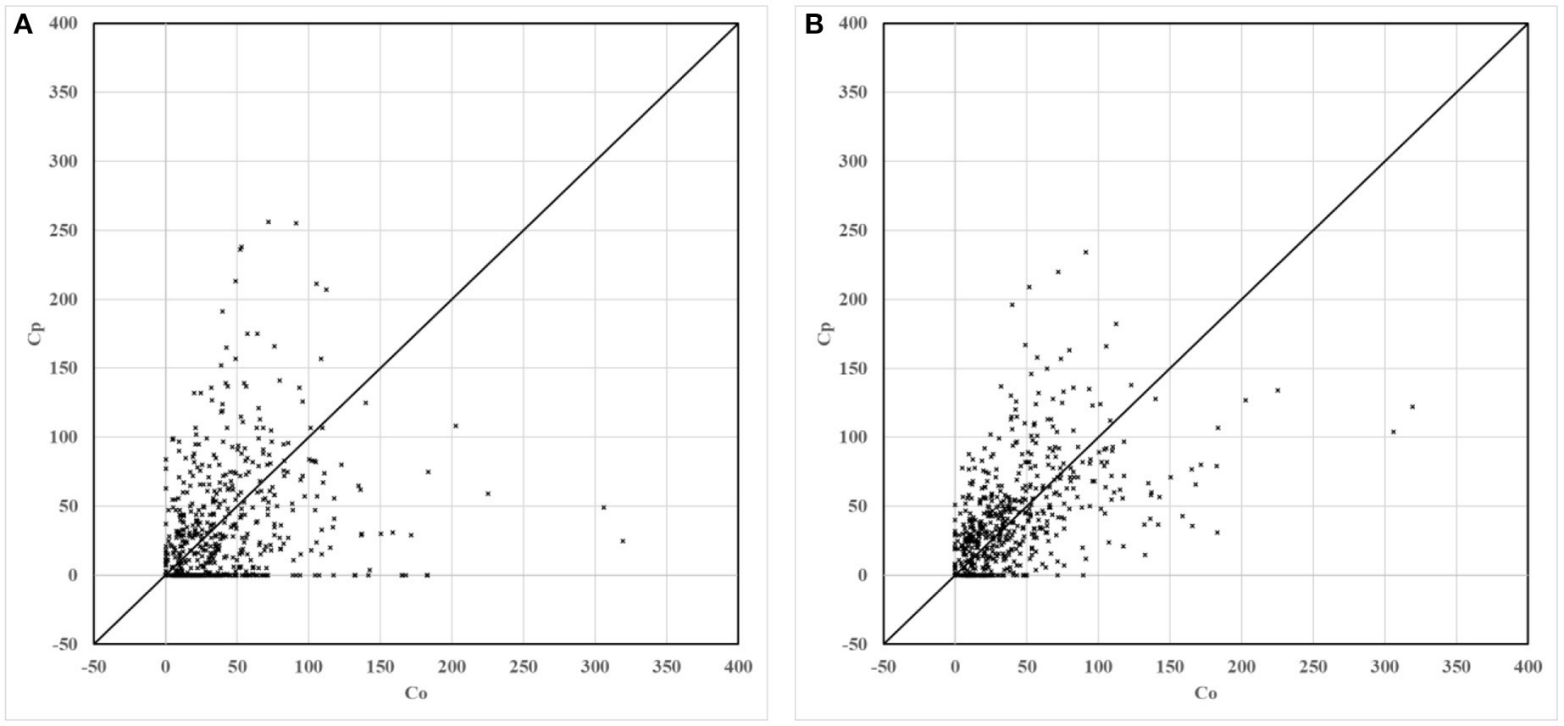
Scatter plots of non-modified and modified model predictions vs. observations. **(A)** Non-modified model predictions vs. observations. **(B)** Modified model predictions vs. observations.

**Figure 6:**
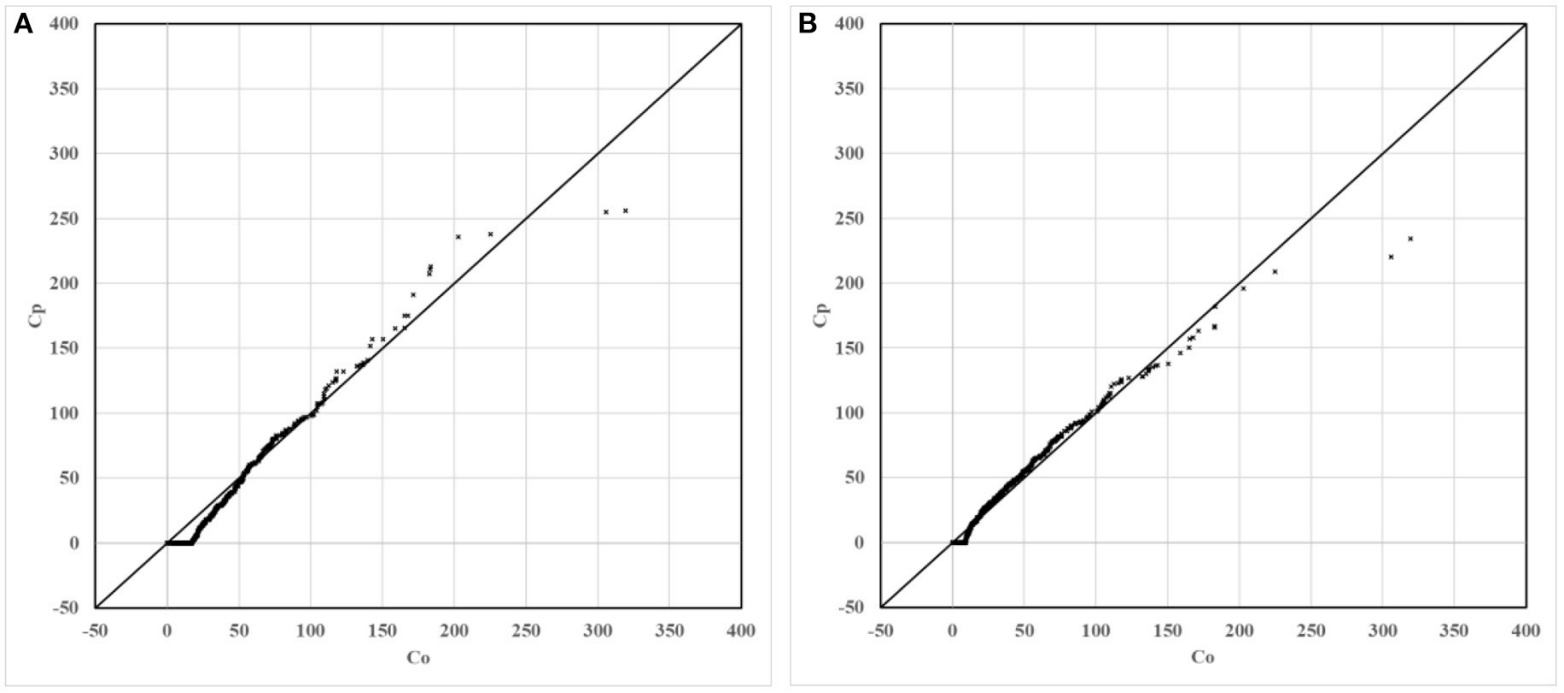
Quantile plots of non-modified and modified model predictions vs. observations. **(A)** Non-modified model predictions vs. observations. **(B)** Modified model predictions vs. observations.

**Figure 7:**
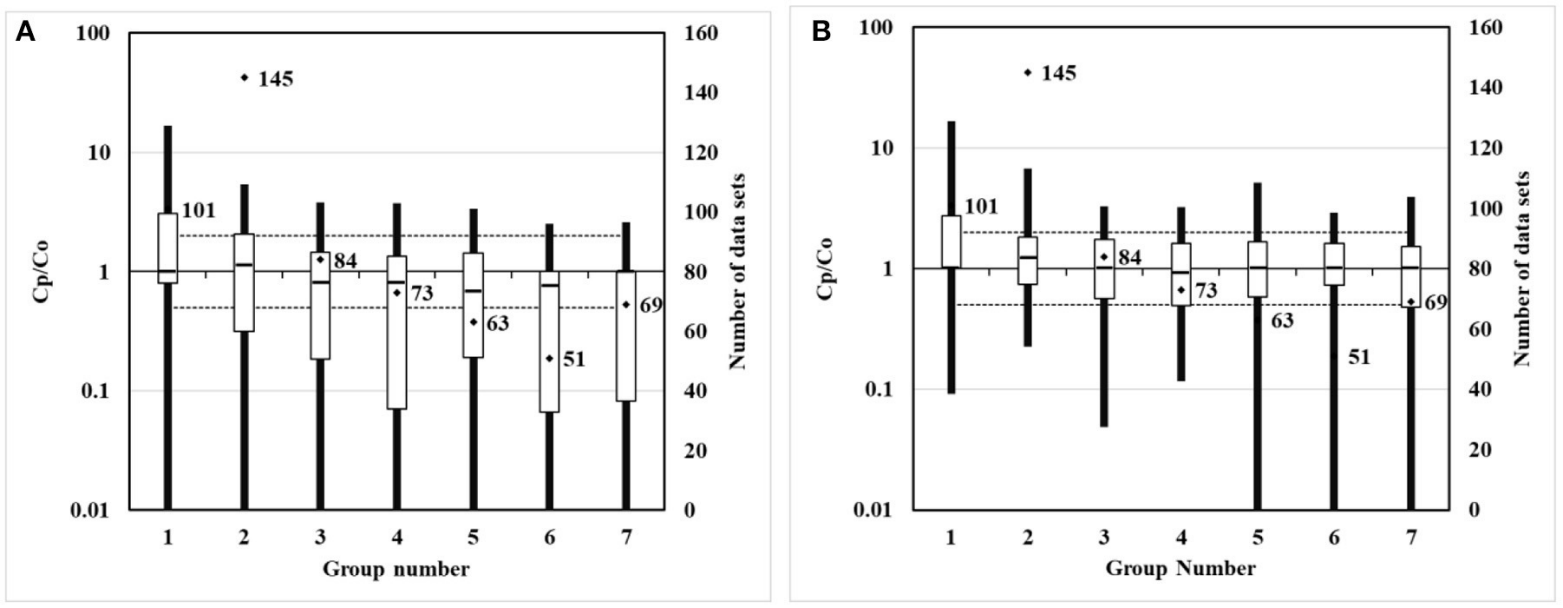
Box plots of non-modified and modified model predictions vs. observations. **(A)** Non-modified model predictions vs. observations. **(B)** Modified model predictions vs. observations.

For the non-modified model and the modified model, scatter, quantile, and box plots of predictions to the observations are drawn, respectively, as shown in Figures 5–**7**. In these figures, Figure (A) represents the plot of the non-modified model, while Figure (B) represents that of the modified model.

For scatter plots shown in Figure 5, 586 groups of observation values are marked with corresponding predicted values in ascending order. In the quantile plot, 586 groups of observation values and predicted values are marked in ascending order, respectively. In the box plot, 586 groups of the ratio of predicted value to observation value are divided into seven groups according to distance. The distance grouping of box plots is shown in Table 3. The lower edge of the box line is the minimum value in the group, while the upper edge of the box line is the maximum value in the group. The lower edge of the box represents that the value is in the 25% position, while the upper edge of the box represents that the value is in the 75% position. The median of the box represents the value that is in the 50% position.

**Table 3:** Distance grouping corresponding to the box plot.

**Group number**	**Distance/km**	**Number of data sets**
1	0.5, 1, 2	101
2	3, 5	145
3	7	84
4	10	73
5	15	63
6	20	51
7	30, 40, 50	69

As shown in Figure 6, compared to the non-modified model, the scatter plot and the quantile plot of the modified model are closer to the y = x line, and most of the simulation results are in good agreement with the observation results. The prediction performance of the modified model has improved after modification.

In the box plots shown in Figure 7, the modified model median of the box reduces to be closer to 1 in groups 1 and 2, that is, the distance is 0–5 km, while the modified model median of the box increases to be closer to 1 in groups 3–7, that is, the distance is 7–50 km. The Kincaid dataset validation in MVK shows that, compared to values of the non-modified model, the values of the modified model show an enhancement effect beyond 5 km, modulating the prediction values to be closer to the observation values. This development is consistent with the modification effects of mixed layers.

#### 3.2.2. Statistical analysis of simulation results

The non-modified diffusion model and the modified diffusion model are shown in Table 4, with the observation values as shown in Table 4. The closer the statistical indicators are to the observation values, the higher the accuracy of the model.

**Table 4:** Comparison table of the statistical results for models' simulation data.

**Model**	**Mean**	**Sigma**	**FB**	**NMSE**	**FA2**
Observations	40.96	39.27	0.00	0.00	1.00
Modified model	42.36	38.57	−0.03	0.87	0.55
Non-modified model	35.00	43.46	0.20	1.79	0.35

The mean value of the non-modified model is 35, while the mean value of the modified model increases to 42.36, which is closer to the observation value. The non-modified model does not consider the mixed layer modification, ignoring the inhibition effect of the mixed layer on the results pertaining to atmospheric diffusion. The non-modified model has a tendency to underestimate the results pertaining to atmospheric diffusion, so the prediction results of the non-modified model turn out to be smaller than the observation results. The correlation coefficient of the non-modified model is 0.26, while the correlation coefficient of the modified model reaches 0.5, indicating that the prediction results of the modified model are more consistent with the observation values.

As shown in Table 4, the non-modified model mean square error (NMSE) is 1.79, which is larger than the observation value. The FA2 factor accounts for a value of about 0.35, which is relatively smaller than the observation value. The modified model FB reduces to −0.03, the NMSE reduces to 0.87, and the FA2 increases to 0.55, indicating that the reliability of the modified model has improved.

## 4. Conclusions

The present study analyzes the atmospheric diffusion model of radionuclides under complex meteorological conditions. Based on the Gaussian atmospheric diffusion model, a radionuclide atmospheric diffusion modified model is established, considering both the inversion temperature and the radionuclide decay modification. The inhibition effect of inversion temperature capping on radionuclide diffusion is modified in the vertical direction. The intensity of the radionuclide source is modified by the decay constant.

The radionuclide atmospheric diffusion modified model is verified. For the chimney height of 180 m, the enhancement effect of the mixed layer on radionuclide concentration is reflected at 5 km for a mixed layer height of 400 m. The decay of radionuclides has a major influence on radionuclides with a smaller half-life, but it has almost no effect on radionuclides with a longer half-life.

The MVK is introduced to validate the modified model for evaluating its prediction performance. The plot shows that, compared with the non-modified model, the values of the modified model show an enhancement effect beyond 5 km, modulating the prediction values to be closer to the observation values. This development is consistent with the modification effects of mixed layers. The statistical indicators of the modified model indicate that the reliability of the modified model has improved. The overall prediction performance of the model is good.

The Gaussian-based modified atmospheric diffusion model presents the advantage of a rapid evaluation of the concentration of radionuclides, thereby providing technical support for the assessment of the consequences of any nuclear accident emergency.

## Data availability statement

The raw data supporting the conclusions of this article will be made available by the authors, without undue reservation.

## Author contributions

TL conceived of the presented idea. TL and XZ developed the theory and performed the computations. TL and SY verified the analytical methods. TL and JW wrote the manuscript with support from JC and JL. All authors discussed the results and contributed to the final manuscript.
